# Annular choroidal detachment following intravitreal aflibercept injection in a patient with nivolumab treatment: a case report

**DOI:** 10.1186/s12886-022-02714-2

**Published:** 2022-12-08

**Authors:** Maho Sato, Hirohisa Kubono, Kazuya Yamashita, Takashi Nagamoto, Yoshiko Ofuji, Saki Sakakura, Ryuki Fukumoto, Seiichiro Hata, Mari Kawamura, Kotaro Suzuki

**Affiliations:** 1 Department of Ophthalmology, Keiyu Hospital, 3-7-3 Minatomirai, Nishiku, Yokohama, Kanagawa Japan; 2 Department of Ophthalmology, Keio University, Tokyo, Japan; 3 Yokohama Sky Eye Clinic, Yokohama, Kanagawa Japan

**Keywords:** Immune checkpoint inhibitor, PD-1, Choroidal detachment, Vogt-Koyanagi-Harada disease, Anti-VEGF antibody

## Abstract

**Background:**

To present a novel case that developed annular choroidal detachment after intravitreal anti-vascular endothelial growth factor antibody injection in a patient after immune checkpoint inhibitor treatment.

**Case presentation:**

A 58-year-old Japanese man presented visual impairment in the right eye. Ophthalmological examination revealed macular edema in the right eye, which suggested the possibility of age-related macular degeneration. Following the intravitreal aflibercept injection, the annular choroidal detachment was observed in the injected eye. As hypotony or thick sclera was not observed, choroidal detachment seemed to have appeared due to enhanced inflammation by intravitreal injection. The patient had a history of stage IV paranasal cavity cancer and was treated with nivolumab, an immune checkpoint inhibitor. The immune response might have been enhanced due to the use of nivolumab so that intravitreal injection triggered inflammation. Three weeks after sub-tenon injection of triamcinolone acetonide, macular edema and choroidal detachment improved.

**Conclusions:**

Intravitreal aflibercept injection caused annular choroidal detachment in our patient, presumably because the immune system was activated after nivolumab treatment. To the best of our knowledge, this is the first case report of annular choroidal detachment that developed after intravitreal injection in a patient with a history of nivolumab therapy. With the increasing use of immune checkpoint inhibitors in patients with various cancers, clinicians should be aware of these potentially associated immune-related adverse events.

## Background

Immune checkpoint inhibitors (ICIs) are immunological agents that prevent cancer cell growth by blocking inhibitory receptors, such as programmed death 1 (PD-1) and its ligand, programmed death protein ligand 1 (PD-L1) [1].

Nivolumab is an ICIs and antibody to PD-1. Ocular side effects secondary to the use of nivolumab are rare and reported to be < 1% of all toxicities, with dry eye and uveitis being the most frequent [1]. The characteristics of ocular side effects have gradually been recognized, and some cases of uveitis after ICI use have been reported to resemble Vogt-Koyanagi-Harada (VKH) disease [2–5].

Herein, we report a unique case of a patient who underwent nivolumab treatment, whose annular choroidal detachment developed following intravitreal injection of anti-vascular endothelial growth factor (VEGF) antibody.

## Case presentation

A 58-year-old Japanese man visited an ophthalmology clinic in May 2020 complaining of visual loss in his right eye, which he had noticed 3 weeks before.

**Fig. 1:**
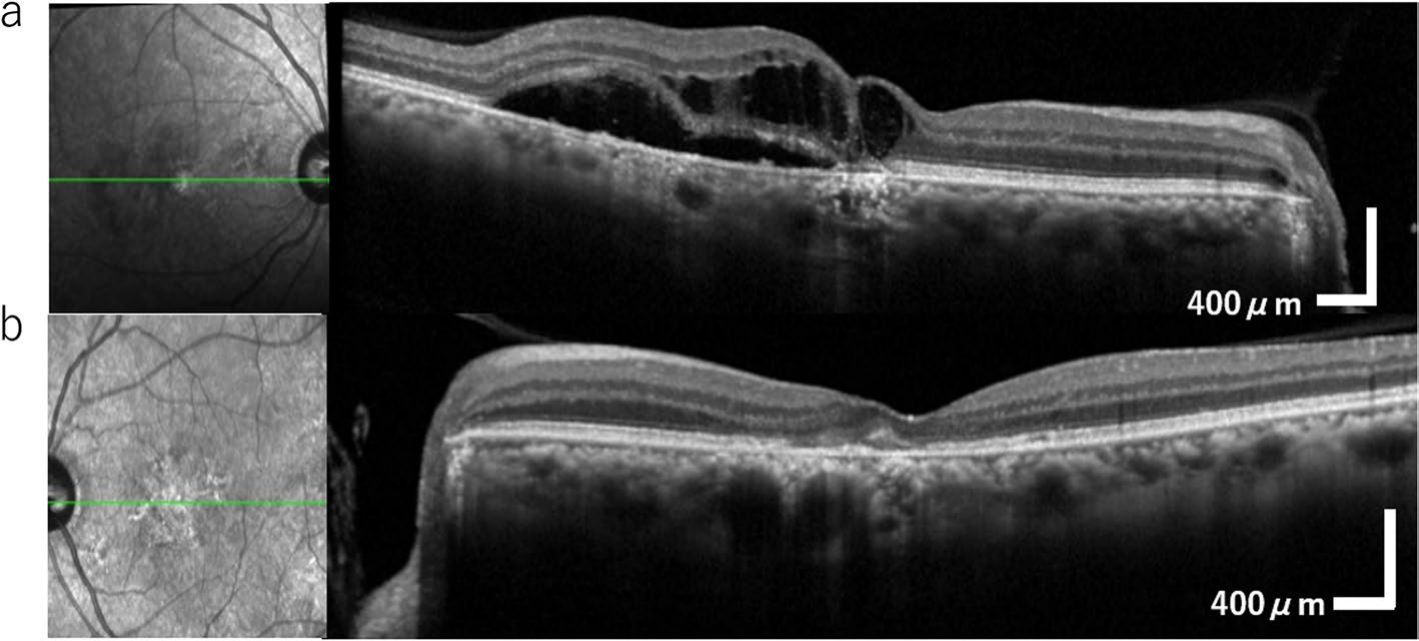
Color fundus photographs (TRC-50DX, Topcon, Japan) were taken at the first visit to the ophthalmology clinic. **a** Macular edema and subretinal fluid involving the macula can be observed in the right eye. **b** No exudative changes are observed in the left eye. An irregular ellipsoid zone is observed in both eyes

The best-corrected visual acuity (BCVA) was 20/50 in the right eye, 20/40 in the left eye, with a refraction of − 0.75 diopter sphere (DS) with − 1.00 diopter cylinder (DC) at 60° in the right eye, − 1.75 DS with − 0.75 DC at 100° in the left eye. The intraocular pressure for the right eye and the left eye was 15 mmHg and 16 mmHg, respectively. Ophthalmologic examination revealed macular edema and subretinal fluid in his right eye (Fig. 1a), as well as disruption of the ellipsoid zone in both eyes (Fig. 1a, b).

In February 2018, the patient was seen at the same clinic for a diabetic retinopathy checkup with HbA1c 10.9%. Visual acuity was 20/20 in both eyes with a refraction of − 0.5 DS with − 1.25 DC at 105° in the right eye, − 1.5 DS with − 0.5 DC at 100° in the left eye. Fundus examination revealed simple diabetic retinopathy in both eyes, with some dot hemorrhage. Macular edema or subretinal fluid was not found; however, optic coherence tomography (OCT) showed disruption in the ellipsoid zone in both eyes; therefore, the patient was followed up for age-related macular degeneration (AMD).

**Fig. 2:**
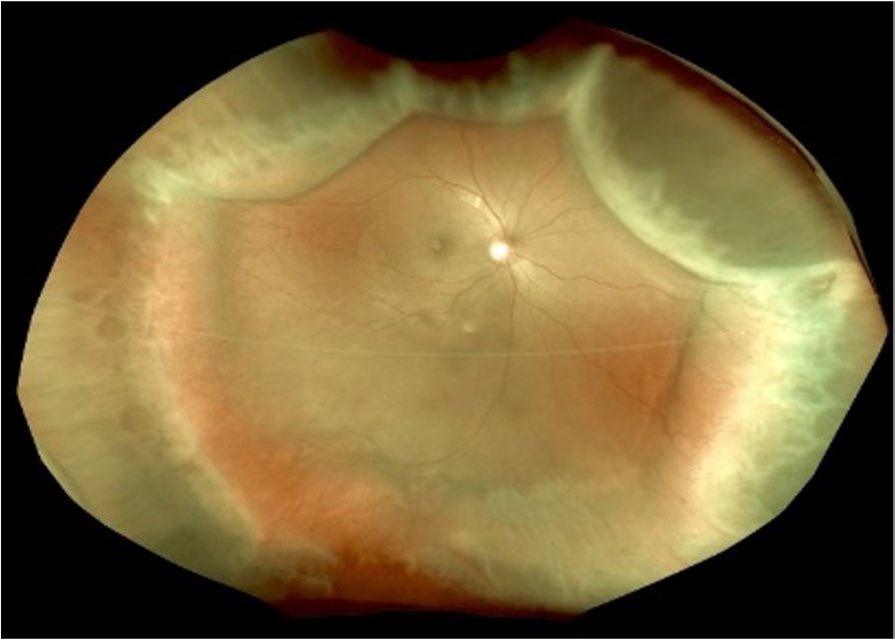
A color fundus photograph (California, Optos, United Kingdom) of the right eye 1 day after intravitreal injection showing 360° choroidal detachments

The exudative change in his right eye was estimated to result from deteriorated AMD, and an intravitreal injection of aflibercept was administered for the first time. On the day following the injection, macular edema worsened, and choroidal detachment was found in all four quadrants of the right eye (Fig. 2). The patient was referred to our hospital for further evaluation.

**Fig. 3:**
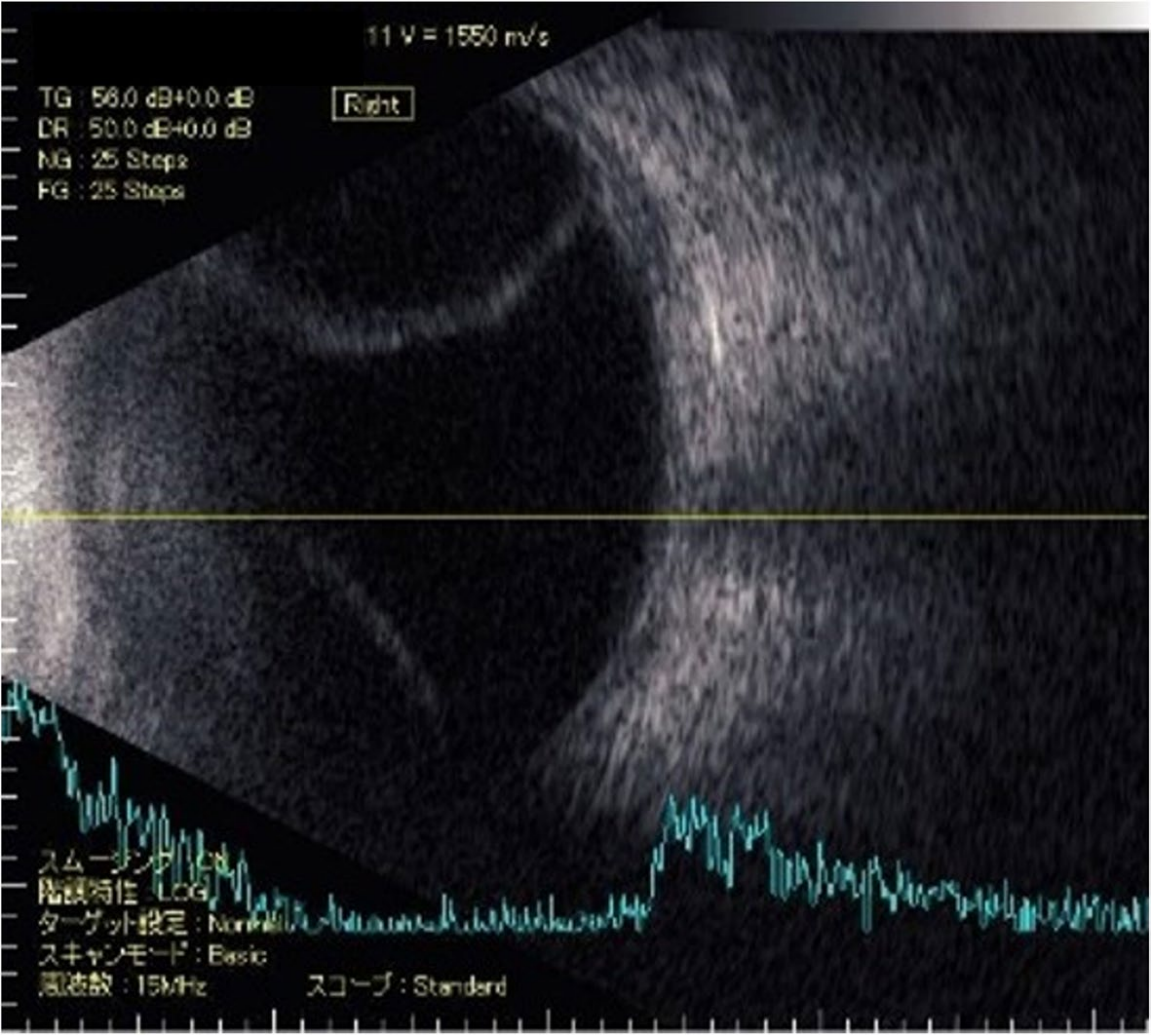
Color fundus photographs (TRC-50DX, Topcon, Japan) when visiting our clinic (**a**, **b**). FA (**c**, **d**) and IA (**e**, **f**) of the early phase. **a**, **b** Color fundus photographs show yellowish lesions after RPE damage in both eyes. FA demonstrates multiple small leaks from the fundus in the right eye (**c**) and window defects in the macula in the left eye (**d**). IA shows delayed choroidal perfusion in the early angiographic phase in both eyes (**e**, **f**). The IA in the left eye (**f**) shows hypofluorescent areas in the macula

On the first visit to our hospital in June 2020, the BCVA was 20/40 in the right eye and 20/25 in the left eye. The intraocular pressure was 13 mmHg in the right eye and 14 mmHg in the left eye. Inflammation was observed in the anterior chamber in both eyes, with a few mutton fat keratic precipitates in the right eye. The fundus examination revealed macular edema and subretinal fluid involvement of the fovea in the right eye (Fig. 3a), with 360° choroidal detachment. Yellowish spots were seen at the fovea in the left eye (Fig. 3b). OCT revealed a damaged outer photoreceptor layer in both eyes.

**Fig. 4:**
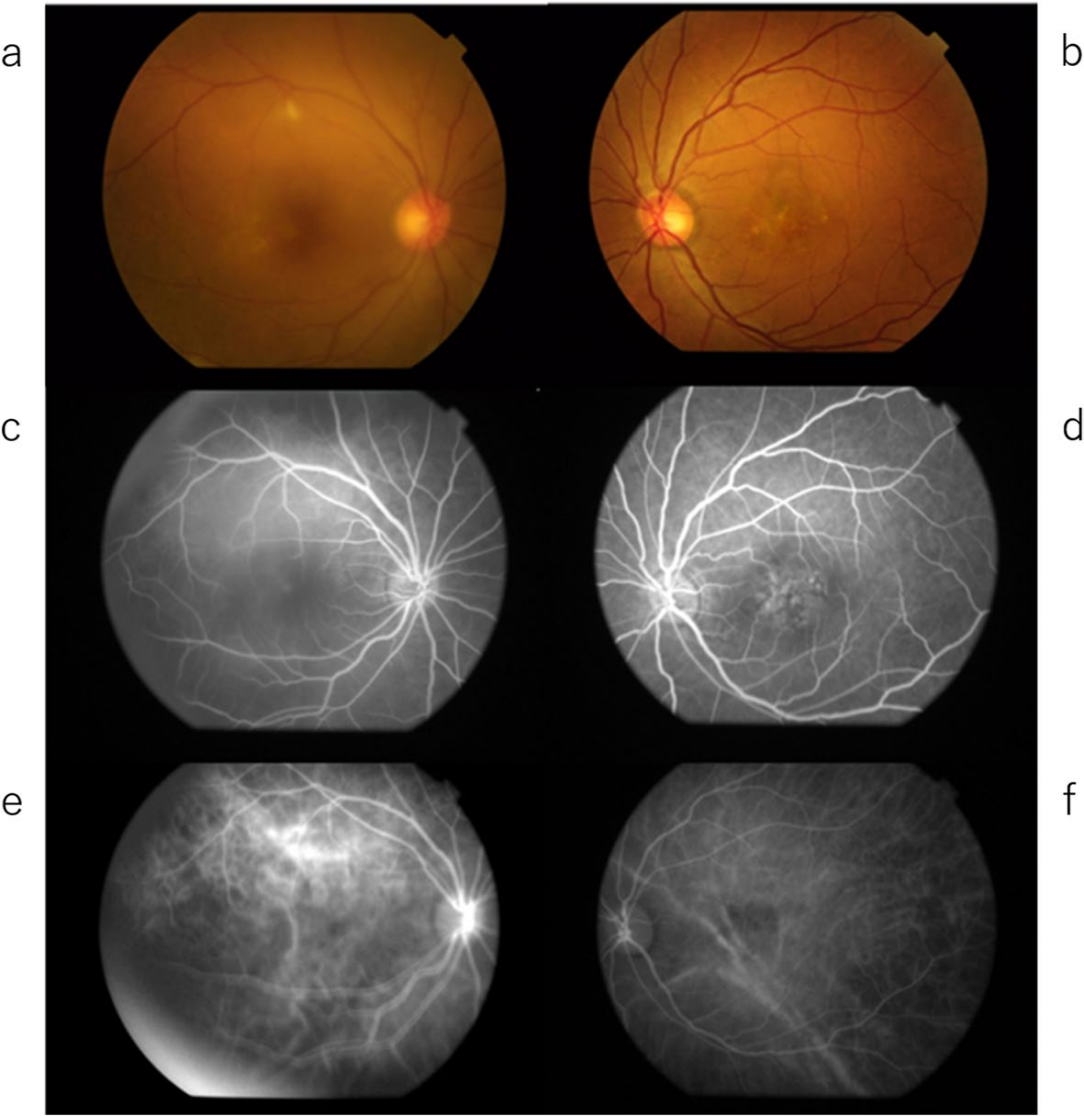
FA (**a**, **b**) and IA (**c**, **d**) in the middle to late phase. Mild leakages are observed from the optic disc and peripheral retinal vessels over the detached choroid in the right eye (**a**). Dark spots are observed in both eyes on IA (**c**, **d**)

**Fig. 5:**
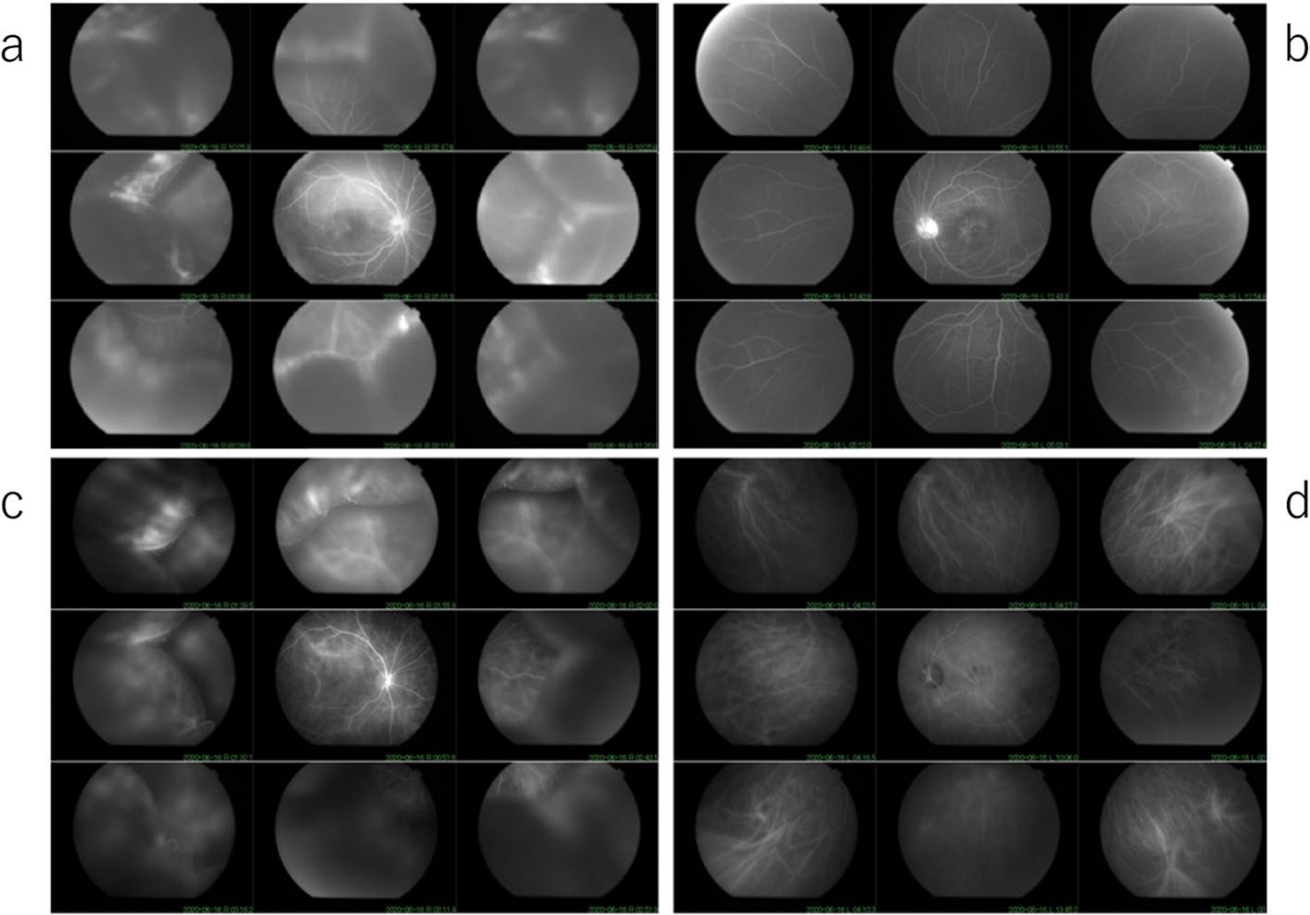
FAF (Triton, Topcon, Japan) shows hypofluorescent areas in both eyes (**a**, **b**)

Fluorescein angiography (FA) revealed multiple leakages of dye in the posterior pole from the early to middle phase and mild dye pooling in the subretinal space in the macular area of the right eye (Fig. 3c and Fig. 4a). Mild leakage from the optic disc and peripheral retinal vessels over the detached choroid was also noted in the right eye (Fig. 4a). On indocyanine green angiography (IA), patchy hypofluorescence was observed during the early angiographic phase in the right eye, suggesting poor choroidal circulation (Fig. 3e and Fig. 4c). Fundus autofluorescence (FAF) showed hypofluorescent areas in the macula, which implicated damaged retinal pigment epithelium (RPE) corresponding to hyperfluorescent areas with leakage in FA (Fig. 5a).

In the left eye, FA showed a window defect in the macula (Fig. 3d and Fig. 4b), which was shown as a hypofluorescent area on IA and FAF, indicating damaged RPE (Fig. 3f, Fig. 4d and Fig. 5b), respectively.

**Fig. 6:**
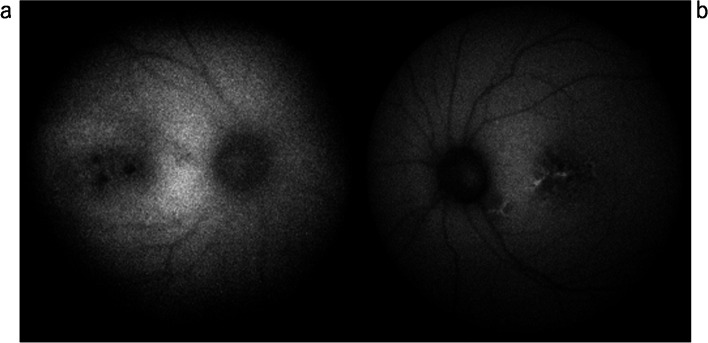
B-mode ultrasound image (UD-8000, TOMEY CORPORATION, Japan) showing choroidal detachment of the right eye. No sign of thick sclera or cancer invasion

B-mode ultrasonography confirmed choroidal detachment in the right eye without abnormal scleral thickness (Fig. 6).

Laboratory data of the patient were within normal range except for HbA1c 7.8% due to diabetes mellitus, which he had been treated with an oral hypoglycemic agent for 10 years.

Through an interview at our hospital, it was revealed that he had a history of stage IV right paranasal cavity tumor, which was diagnosed in 2010. He did not initially request any treatment; however, as the tumor gradually enlarged, the patient underwent radiotherapy and chemotherapy with cisplatin in 2017. As these treatments were ineffective, the patient underwent nivolumab therapy from August 2019 to March 2020, which was discontinued due to ineffectiveness. Computed tomography (CT) and B-mode ultrasonography showed no signs of tumor invasion in the ocular cavity or globe.

Choroidal neovascularization was not found in FA or IA, which suggests that AMD was not the cause of the initial macular edema and subretinal fluid. Instead, angiography findings taken after intravitreal injection, such as multiple leakages in FA and delay in choroidal circulation in IA, resembled the characteristics of VKH. Considering some reports that nivolumab causes VKH-like uveitis, the initial exudative change in the right eye and anterior inflammation in both eyes were due to uveitis after nivolumab treatment. The fact that anterior inflammation existed also in the left eye suggests that inflammation after nivolumab use continued until aflibercept injection, and the patient a was vulnerable to intravitreal injection.

Since choroidal detachment occurred a day after intravitreal injection and there was no sign of uveal effusion or hypotony, we suspected that choroidal detachment developed due to enhanced inflammation by intravitreal injection. In addition, annular choroidal detachment can be observed in VKH cases with enhanced inflammation, which supports the possibility that the present case had VKH-like uveitis as a background.

**Fig. 7:**
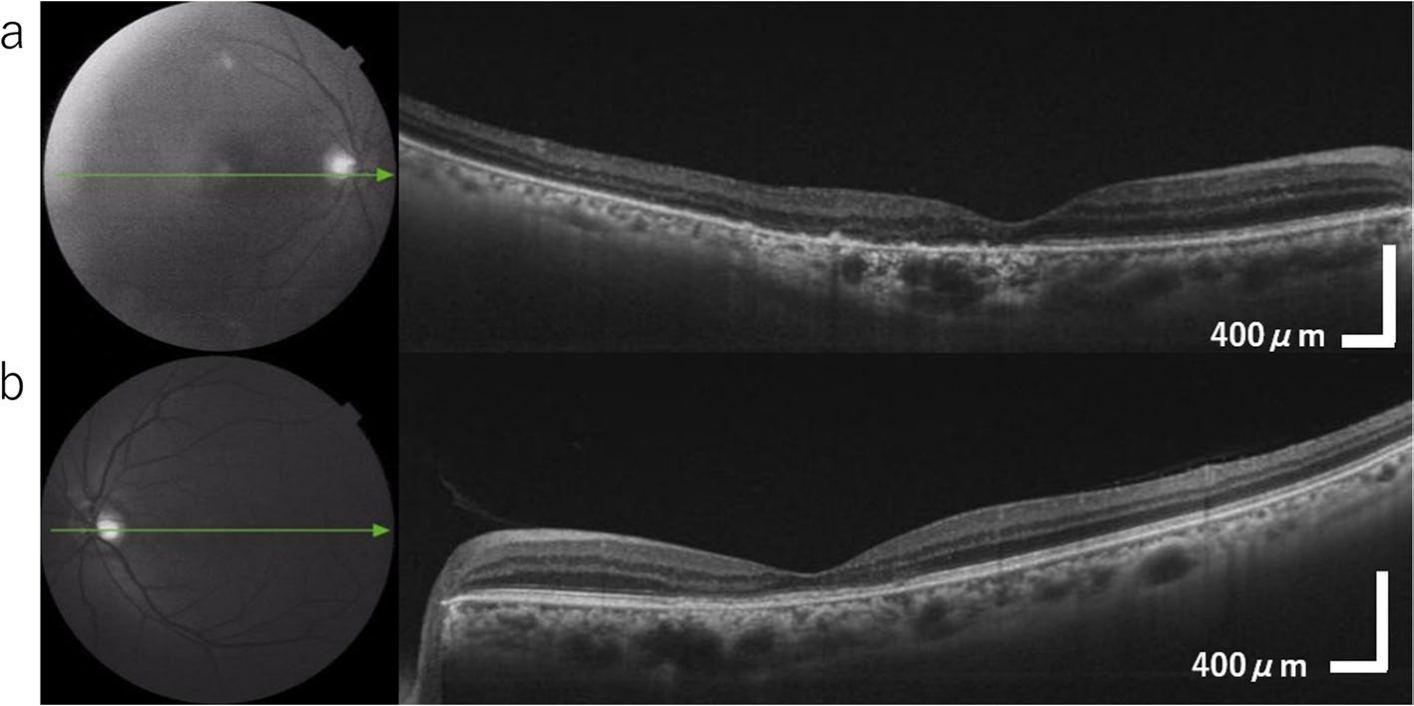
A fundus OCT (Swept Source OCT, Triton, Topcon, Japan) after STTA. **a** Exudative change has settled in the eye. **b** No exudative changes are observed in the left eye

In previously reported cases, corticosteroid treatment was effective for VKH-like uveitis after nivolumab use [2]. We started topical corticosteroid treatment in both eyes, which was effective for anterior inflammation in both eyes but not for posterior changes in the right eye. We then administered a sub-tenon injection of triamcinolone acetonide (STTA, 10 mg). Three weeks after STTA, the choroidal detachment disappeared in all quadrants as well as in the subretinal fluid, macular edema, and anterior inflammation (Fig. 7a). There was no change in the macula in the left eye (Fig. 7b). Visual acuity was 20/50 in the right eye and 20/22 in the left eye. Although the inflammation was resolved, visual acuity in the right eye did not improve due to progressive posterior subcapsular cataracts after inflammation.

In December 2020, the patient was monitored for 6 months after STTA without additional treatment, and the inflammation had not recurred.

## Discussion and conclusions

This is a case of annular choroidal detachment that occurred after intravitreal injection of an anti-VEGF antibody for macular edema.

### The cause of choroidal detachment

When we consider the cause of choroidal detachment, without signs of hypotony or uveal effusion, we suspected that the reason for choroidal detachment was enhanced inflammation by intravitreal injection of aflibercept.

A similar case of choroidal detachment after intravitreal injection has been previously reported. It was a VKH case where inflammation had settled for several years before choroidal neovascularization appeared [6]. In this case, annular choroidal detachment developed 2 days after intravitreal anti-VEGF injection. Similar to VKH disease, patients develop choroidal detachment when there is enormous inflammation, and choroidal inflammation increases permeability of the choroidal vessels into the extravascular space [7, 8].

Sterile intraocular inflammation is one of the side effects of aflibercept [9]. Patients may be more prone to inflammation when breakdown of the blood-retina barrier occurs in certain conditions compromising the immune privilege of the vitreous [10] and history of inflammation [11]. Our case differs from typical sterile intraocular inflammation cases, since our case had relatively silent anterior chamber inflammation and no vitreous inflammation, while having 360°choroidal detachment. However, our case might have had similar compromised immune system as VKH-like uveitis.

Considering the fact that, to our knowledge, there has been only a single documented case of choroidal detachment after intravitreal injection and that the patient has a history of VKH, it can be estimated that patients with VKH might be immunologically sensitive to intravitreal injection of aflibercept. In patients with VKH, the immune system against melanocytes is already formed so that intravitreal injection might trigger inflammation.

In our patient, the angiographic results after intravitreal injection resembled the findings in VKH, and the decrease in choroidal thickness in the right eye after STTA supported that there had been inflammation at the choroid, similar to VKH.

After choroidal detachment occurred, choroidal thickness was 370 μm in the right eye and 296 μm in the left eye. The axial length was measured by IOL Master 500 (Carl Zeiss Meditec AG, Jena, Germany) as 23.52 mm in the right eye and 23.45 mm in the left eye. The refraction was − 3.0 DS with − 1.0 DC at 80° in the right eye and − 2.0 DS with − 1.5 DC at 100° in the left eye. Refraction after treatment was − 0 DS with − 1.0 DC at 80° in the right eye and − 1.0 DS with − 1.5 DC at 100° in the left eye, indicating the recovery of the myopic change induced by ciliary inflammation. The choroidal thickness in August 2020 decreased to 273 μm on the right and 263 μm on the left.

The myopic shift, changes in choroidal thickness, and anterior inflammation in both eyes suggest that uveitis before aflibercept injection had occurred in both eyes, although macular edema and subretinal fluid were only observed in the right eye.

Consequently, our patient developed VKH-like uveitis after nivolumab treatment, and choroidal detachment occurred after intravitreal injection. Treatment with nivolumab could have been a reason for immune sensitivity; however, we cannot be sure whether the procedure of intravitreal injection of nivolumab or antiVEGF triggered choroidal detachment.

### The cause of macula edema

If nivolumab has influenced the immune system in this patient, it is also reasonable to think that nivolumab caused initial macular edema and subretinal fluid; however, other differential diagnoses must be considered to produce exudative changes in the macula.

OCT since 2018 showed macular degeneration in both eyes; therefore, the patient was followed up for AMD. However, choroidal neovascularization was not detected in FA after intravitreal injection, and there was less possibility of AMD causing macular edema.

Multiple RPE damage can be attributed to central serous chorioretinopathy (CSC) or multifocal posterior pigment epitheliopathy (MPPE) possibly happened in the past; however, we cannot be sure whether the exudative change at this time is due to CSC or MPPE, as these diseases are not usually accompanied by inflammation in the anterior chamber.

Considering that this patient had a history of cancer and radiotherapy, bilateral diffuse uveal melanocytic proliferation (BDUMP) and radioretinopathy were also differential diagnoses for macular edema; however, there were no characteristic findings of these diseases in the fundus.

Diabetic retinopathy can also cause macular edema, which cannot be ruled out based on the findings in this patient.

None of the differential diagnoses above are decisive, and we cannot deny the possibility that macular edema was caused by nivolumab. However, regardless of the cause of the initial macular edema, there must be a foundation that enables strong inflammation to occur after intravitreal injection, and that foundation might be nivolumab treatment.

There are two things in our case that are unusual for uveitis after using ICI.

First, when we consider the timing of the occurrence of adverse events, most of the reported PD-1 inhibitor-related ocular complications occurred within a few weeks to months after infusion [12], and our patient noticed vision loss 3 months after the last treatment. Osa et al. reported that prolonged nivolumab binding was detected more than 20 weeks after the last infusion [13], which supports that logically there is a possibility that macula edema in our patient was caused by nivolumab. For the same reason, it is reasonable that anterior inflammation occurred by aflibercept and was seen in both eyes at the first visit to our hospital.

In addition, many reported cases had bilateral uveitis. Our patient had exudative changes in the macula only in the right eye; however, there have been some cases of lateral uveitis due to ICI [14]. In addition, inflammation in the anterior chamber and dark spots on the IA in the left eye suggest that although there was a difference in the severity of inflammation in each eye, we can say that our patient had bilateral VKH-like uveitis.

In summary, this is a case in which choroidal detachment occurred after intravitreal injection. The injection triggered enormous choroidal inflammation as the immune system was activated after nivolumab treatment.

## Conclusion

Herein, we describe a case of choroidal detachment after intravitreal injection of an anti-VEGF antibody. To the best of our knowledge, this is the first case report of annular choroidal detachment that developed after intravitreal injection in a patient with a history of nivolumab therapy. This case suggests that intravitreal injection might cause ocular inflammation with an underlying immune sensitivity, such as VKH and VKH-like uveitis, after ICI treatment. With the increasing number of cancers for which ICIs are used and a growing number of anti-VEGF uses for various ocular conditions, it is important for ophthalmologists to be aware of the application and side effects of anti-VEGF.

## Data Availability

Not applicable.

## Abbreviations

ICIs: Immune checkpoint inhibitors
PD-1: Programmed death 1
PD-L1: Programmed death protein ligand 1
VKH: Vogt-Koyanagi-Harada disease
VEGF: Vascular endothelial growth factor
BCVA: Best-corrected visual acuity
DS: Diopter sphere
DC: Diopter cylinder
OCT: Optic coherence tomography
AMD: Age-related macular degeneration
FA: Fluorescein angiography
IA: Indocyanine green angiography
RPE: Retinal pigment epithelium
CT: Computed tomography
HLA: Human leukocyte antigen
STTA: Sub-tenon injection of triamcinolone acetonide
CSC: Central serous chorioretinopathy
MPPE: Multifocal posterior pigment epitheliopathy
BDUMP: Bilateral diffuse uveal melanocytic proliferation

## Declarations

### Concent for pubication

Written consent to publish this information was obtained from the patient.
